# Enhancing prostate cancer diagnosis and reducing unnecessary biopsies with [^18^F]DCFPyL PET/CT imaging in PI-RADS 3/4 patients

**DOI:** 10.1038/s41598-024-65452-z

**Published:** 2024-07-05

**Authors:** Yang Fu, Min Zhao, Jie Chen, Qiang Wen, Bin Chen

**Affiliations:** Department of Nuclear Medicine, China-Japan Union Hospital of Jilin University, No. 126, Xiantai Street, Changchun, 130033 China

**Keywords:** Prostate cancer, [^18^F]DCFPyL PET/CT, PI-RADS, mpMRI, Biopsy, Urological cancer, Medical research

## Abstract

For patients presenting with prostate imaging reporting and data system (PI-RADS) 3/4 findings on magnetic resonance imaging (MRI) examinations, the standard recommendation typically involves undergoing a biopsy for pathological assessment to ascertain the nature of the lesion. This course of action, though essential for accurate diagnosis, invariably amplifies the psychological distress experienced by patients and introduces a host of potential complications associated with the biopsy procedure. However, [^18^F]DCFPyL PET/CT imaging emerges as a promising alternative, demonstrating considerable diagnostic efficacy in discerning benign prostate lesions from malignant ones. This study aims to explore the diagnostic value of [^18^F]DCFPyL PET/CT imaging for prostate cancer in patients with PI-RADS 3/4 lesions, assisting in clinical decision-making to avoid unnecessary biopsies. 30 patients diagnosed with PI-RADS 3/4 lesions through mpMRI underwent [^18^F]DCFPyL PET/CT imaging, with final biopsy pathology results as the “reference standard”. Diagnostic performance was assessed through receiver operating characteristic (ROC) analysis, evaluating the diagnostic efficacy of molecular imaging PSMA (miPSMA) visual analysis and semi-quantitative analysis in [^18^F]DCFPyL PET/CT imaging. Lesions were assigned miPSMA scores according to the prostate cancer molecular imaging standardized evaluation criteria. Among the 30 patients, 13 were pathologically confirmed to have prostate cancer. The sensitivity, specificity, positive predictive value, negative predictive value, and accuracy of visual analysis in [^18^F]DCFPyL PET/CT imaging for diagnosing PI-RADS 3/4 lesions were 61.5%, 88.2%, 80.0%, 75.0%, and 76.5%, respectively. Using SUVmax 4.17 as the optimal threshold, the sensitivity, specificity, positive predictive value, negative predictive value, and accuracy for diagnosis were 92.3%, 88.2%, 85.7%, 93.8%, and 90.0%, respectively. The area under the ROC curve (AUC) for semi-quantitative analysis was 0.94, significantly higher than visual analysis at 0.80. [^18^F]DCFPyL PET/CT imaging accurately diagnosed benign lesions in 15 (50%) of the PI-RADS 3/4 patients. For patients with PI-RADS 4 lesions, the positive predictive value of [^18^F]DCFPyL PET/CT imaging reached 100%. [18F]DCFPyL PET/CT imaging provides potential preoperative prediction of lesion nature in mpMRI PI-RADS 3/4 patients, which may aid in treatment decision-making and reducing unnecessary biopsies.

## Introduction

Prostate cancer ranks as the second most prevalent malignant tumor among men worldwide, with over 1.41 million new cases and 370,000 deaths annually, posing a significant threat to male health^1^. In recent years, Multiparametric Magnetic Resonance Imaging (mpMRI), owing to its rich imaging parameters, has emerged as a pivotal imaging modality for the initial diagnosis and localization of prostate cancer, enabling comprehensive assessments of prostate tumor lesions from anatomical, vascular, tumor cell density, and invasiveness perspectives^2^. Additionally, for patients with negative biopsy results, mpMRI can enhance prostate cancer detection rates and underscore the importance of Active Surveillance (AS) protocols^3,4^. To standardize and optimize the diagnosis of prostate lesions using mpMRI, PI-RADS was developed to aid in the identification of prostate cancer^5^. PI-RADS stratifies the risk of prostate lesions defined by mpMRI based on the likelihood of patients developing prostate cancer. However, the interpretation of mpMRI images is subject to significant subjectivity in assessing lesion shape, signal intensity, and boundaries, leading to challenges in achieving consistent diagnostic results that meet clinical demands. Especially in cases of PI-RADS 3/4 lesions, the diagnosis of prostate cancer remains ambiguous, and current guidelines recommend that these lesions should undergo biopsy^6^. Nevertheless, research indicates that the positive biopsy rate for PI-RADS 3 lesions ranges from 0.0 to 24.7%, while for PI-RADS 4 lesions, it varies from 22.0 to 64.0%^7–9^. As a invasive procedure, prostate biopsy can result in a range of adverse reactions, with approximately 51% of patients experiencing hematuria, 40% reporting pain, and an increased risk of infection and complications^10,11^. Furthermore, the overdiagnosis of low-risk prostate lesions can impose unnecessary economic and psychological burdens on patients. Currently, the imperative challenge in clinical practice is to develop methods for further distinguishing the nature of prostate lesions in PI-RADS 3/4 patients to reduce the need for unnecessary biopsies.

Prostate-Specific Membrane Antigen (PSMA) is a type II transmembrane glycoprotein secreted by prostate epithelial cells. Its expression in prostate cancer cells is 100 to 1000 times higher than in normal prostate cells, and its expression levels are associated with prostate specific antigen (PSA) levels, the International Society of Urological Pathology (ISUP) grading, and survival rates^12^. Kawada et al.^13^ in comparing the roles of PET/CT and mpMRI in prostate-targeted biopsies, found that PSMA PET-targeted biopsies have higher detection accuracy. [^18^F]DCFPyL, as a second-generation PSMA imaging agent, exhibits high affinity and favorable pharmacokinetics. Previous studies have demonstrated that [^18^F]DCFPyL PSMA PET has similar accuracy and reliability to mpMRI regarding primary prostate cancer localization and extra-prostatic disease. PSMA parameters reaches substantial inter-reader agreement and a significantly higher intra-class correlation (ICC). mpMRI has a lower inter-reader agreement and only moderate-to-good agreement for ICC quantification^14,15^. Therefore, this study explores the diagnostic value of [^18^F]DCFPyL PET/CT imaging in patients with mpMRI PI-RADS scores of 3/4.

## Materials and methods

### Study subjects

A retrospective collection and analysis of clinical and radiological data from suspected prostate cancer patients who were treated at Jilin University China-Japan Union Hospital from June 2021 to April 2023. Inclusion criteria: (1) Prostate mpMRI with a PI-RADS score of 3/4 conducted one month before [^18^F]DCFPyL PET/CT examination. (2) Patients who had not undergone any prostate cancer-related treatments such as surgery, hormonal therapy, or radiation therapy before the imaging examination. (3) Patients who underwent prostate biopsy within two months after the [^18^F]DCFPyL PET/CT examination and obtained precise pathological results. Exclusion criteria: (1) Prostate invasive procedures conducted within one month before the [^18^F]DCFPyL PET/CT examination. (2) Patients with concomitant malignancies. (3) Patients with incomplete clinical data or follow-up records.

### Ethical approval and consent to participate

All ethical and moral issues were considered in this study. Informed consent was obtained from the patients or their close relative. This study was approved by the ethics committee of Jilin University China-Japan Union Hospital (Approval ID: (2020052002). We confirm that all methods were performed in accordance with the relevant guidelines and regulations.

### [^18^F]DCFPyL PET/CT imaging

[^18^F]DCFPyL is prepared by the PET Center of the Department of Nuclear Medicine at Jilin University China-Japan Union Hospital. The radioactive isotope [^18^F] is produced by a medical cyclotron (HM-10, Sumitomo, Japan), and is transferred to a multifunctional automated synthesis module for drug synthesis, achieving a radiochemical purity exceeding 95%. All patients received an elbow venous injection of [^18^F]DCFPyL at a dose of 0.1 mCi/kg, with no special preparation required before injection. PET/CT (uMI780, United Imaging Healthcare, China) imaging was performed 60 min after injection, covering the region from the top of the skull to the mid-thigh. A spiral CT scan was conducted first with the following parameters: tube voltage of 120 kV, tube current of 100 mAs, and a slice thickness of 0.5 mm. Subsequently, PET imaging was performed in the same region, with each bed lasting 2.5 min, totaling 4–5 beds, using an image matrix of 600 × 600 and a slice thickness of 5 mm. In case of urine contamination or urinary residue in the bladder and ureters affecting image interpretation, a diuretic delayed imaging was performed 120 min after the initial imaging. After scanning, PET data underwent attenuation correction using CT, followed by image reconstruction using the ordered subset expectation maximization algorithm (OSEM). All images were transferred to the uWS-CT software (United Imaging Healthcare, China) for co-registration and interpretation (Supplementary Information).

### Image analysis

Image analysis was independently conducted by two experienced nuclear medicine physicians from our hospital using a double-blind approach. Visual analysis was performed using the PROMISE criteria to score prostate cancer lesions (miPSMA). The SUV of liver was measured by placing a 3-cm-diameter circular ROI in the normal inferior right liver lobe in the axial plane; the blood pool, by centering a 2-cm-diameter circular ROI in the aortic arch in the axial plane; the parotid gland, by centering a 1.5-cm-diameter circular ROI in the right parotid gland in the axial plane. A score of 0 indicated uptake lower than the mean blood pool, 1 indicated uptake higher than the mean blood pool but lower than the mean liver uptake, 2 indicated uptake higher than the mean liver uptake but lower than the mean parotid uptake, and 3 indicated uptake higher than the mean parotid uptake. Lesions with a miPSMA score of 2 or higher were considered positive^16^. Additionally, in [^18^F]DCFPyL PET/CT imaging, ROI was delineated around prostate-positive lesions or lesions identified in MR, and the maximum standardized uptake value (SUVmax) of the lesions was measured for semi-quantitative analysis.

### MRI imaging

MRI exams were obtained with a 3.0T system using a pelvic phased-array coil (Skyra, Siemens Healthcare), with the findings reported per PI-RADS version 2. Patients were examined in the supine position. The prostate gland was studied using the T2-weighted MRI, diffusion-weighted MRI and dynamic contrast-enhanced MRI.

### Histopathological examination

All patients underwent 12-core systematic biopsy and targeted biopsy by experienced urological surgeons, combining mpMRI and PET/CT imaging results to ensure accurate correlation with imaging data. The obtained specimens were pathologically assessed by two experienced urologic pathologists according to the 2014 ISUP Gleason grading guidelines.

### Statistical analysis

Data analysis was performed using SPSS 25.0 statistical software. Descriptive statistics for continuous variables were expressed as average value and range, while categorical data was presented as count and ratio. Student’s t-test was employed for comparing normally distributed continuous variables, and Mann–Whitney U test for non-normally distributed variables. We plotted ROC curves based on the SUVmax values obtained from regions of interest delineated on [^18^F]DCFPyL PET/CT images. The optimal diagnostic threshold for SUVmax was determined by calculating the Youden index, and diagnostic sensitivity, specificity, positive predictive value, negative predictive value, and accuracy were calculated. Consistency was assessed using Kappa analysis, with a significance level set at *P* < 0.05.

### Ethics statement

The studies involving human participants were reviewed and approved by the Ethics Committee of China-Japan Union Hosptal of Jilin University. The patients/participants provided their written informed consent to participate in this study.

## Results

### Clinical characteristics and histopathological findings

A total of 30 patients who met the inclusion and exclusion criteria were retrospectively reviewed. The average age of the patients was 69.1 years (range: 57–82 years), with an average total prostate specific antigen (TPSA) level of 17.0 ng/ml (range: 1.61–100 ng/ml). Among them, 11 patients had a PI-RADS score of 3, while 19 patients had a score of 4. All 30 patients underwent transrectal ultrasound-guided pathological examinations. Histopathologically, 13 patients were diagnosed with prostate cancer, with 4 having a Gleason score of 6, 6 with a score of 7, 2 with a score of 8, and 1 with a score of 10. Additionally, 17 patients were diagnosed with benign prostate conditions, with 6 having prostate hyperplasia, 2 having chronic atrophic tissue, 9 having prostate inflammation. Patients in the prostate cancer and benign condition groups showed no significant statistical differences in age and TPSA levels (*P* > 0.05). In the prostate cancer group, patients with a PI-RADS score of 4 accounted for 92.3%, significantly higher than the 41.2% in the benign condition group (*P* < 0.05), as detailed in Table 1.

**Table 1:** Characteristics of the study patients.

Characteristics	Number
Age (year)	69.1 (57–82)
PSA (ng/ml)	17.0 (1.61–100)
mpMRI PI-RADS	
3	11 (33.7%)
4	19 (63.3%)
Prostate cancer	13/30 (43.3%)
Gleason score 6	4 (30.8%)
Gleason score 7	6 (46.1%)
Gleason score 8	2 (15.4%)
Gleason score 9	0 (0%)
Gleason score 10	1 (7.7%)
Benign conditions	17/30 (56.7%)
Prostatitis	9 (52.9%)
Benign prostate hyperplasia	6 (35.3%)
Chronic atrophic tissue	2 (11.8%)

PSA = prostate specific antigen; Gleason score 6 = Gleason score 3 + 3; Gleason score 7 = Gleason score 3 + 4 or 4 + 3; Gleason score 8 = Gleason score 4 + 4; Gleason score 9 = Gleason score 4 + 5 or 5 + 4; Gleason score 10 = Gleason score 5 + 5.

### Visual analysis of [^18^F]DCFPyL PET/CT for prostate cancer

In visual analysis, the prostate cancer group had 5 cases with a miPSMA score of 1, 3 cases with a score of 2, and 5 cases with a score of 3. In the benign conditions group, there were 4 cases with a miPSMA score of 0, 11 cases with a score of 1, 1 case with a score of 2, and 1 case with a score of 3, as detailed in Table 2. We defined a miPSMA score of ≥ 2 as positive, with a sensitivity of 61.5%, specificity of 88.2%, positive predictive value of 80.0%, negative predictive value of 75.0%, and an accuracy of 76.5%. The ROC analysis yielded an AUC of 0.80. The application of miPSMA scoring for diagnosing prostate cancer showed moderate consistency with histopathological results (K = 0.51, *P* < 0.05). There were 5 false-negative lesions, all of which exhibited lower metabolic levels than the liver. However, pathological examination confirmed them as actual prostate cancer cases. There were 2 false-positive lesions, with one exhibiting higher metabolic levels than the salivary glands and the other higher than the liver. However, pathological examination confirmed them as cases of prostate inflammation.

**Table 2:** miPSMA score of [^18^F]DCFPyL PET/CT.

	miPSMA score				*P*
	0	1	2	3	
Benign conditions	4	11	1	1	< 0.05
Prostate cancer	0	5	3	5	

miPSMA score 0 = uptake lower than the mean blood pool; miPSMA score 1 = uptake higher than the mean blood pool but lower than the mean liver uptake; miPSMA score 2 = uptake higher than the mean liver uptake but lower than the mean parotid uptake; miPSMA score 3 = uptake higher than the mean parotid uptake.

### Semi-quantitative analysis of [^18^F]DCFPyL PET/CT for prostate cancer

**Figure 1:**
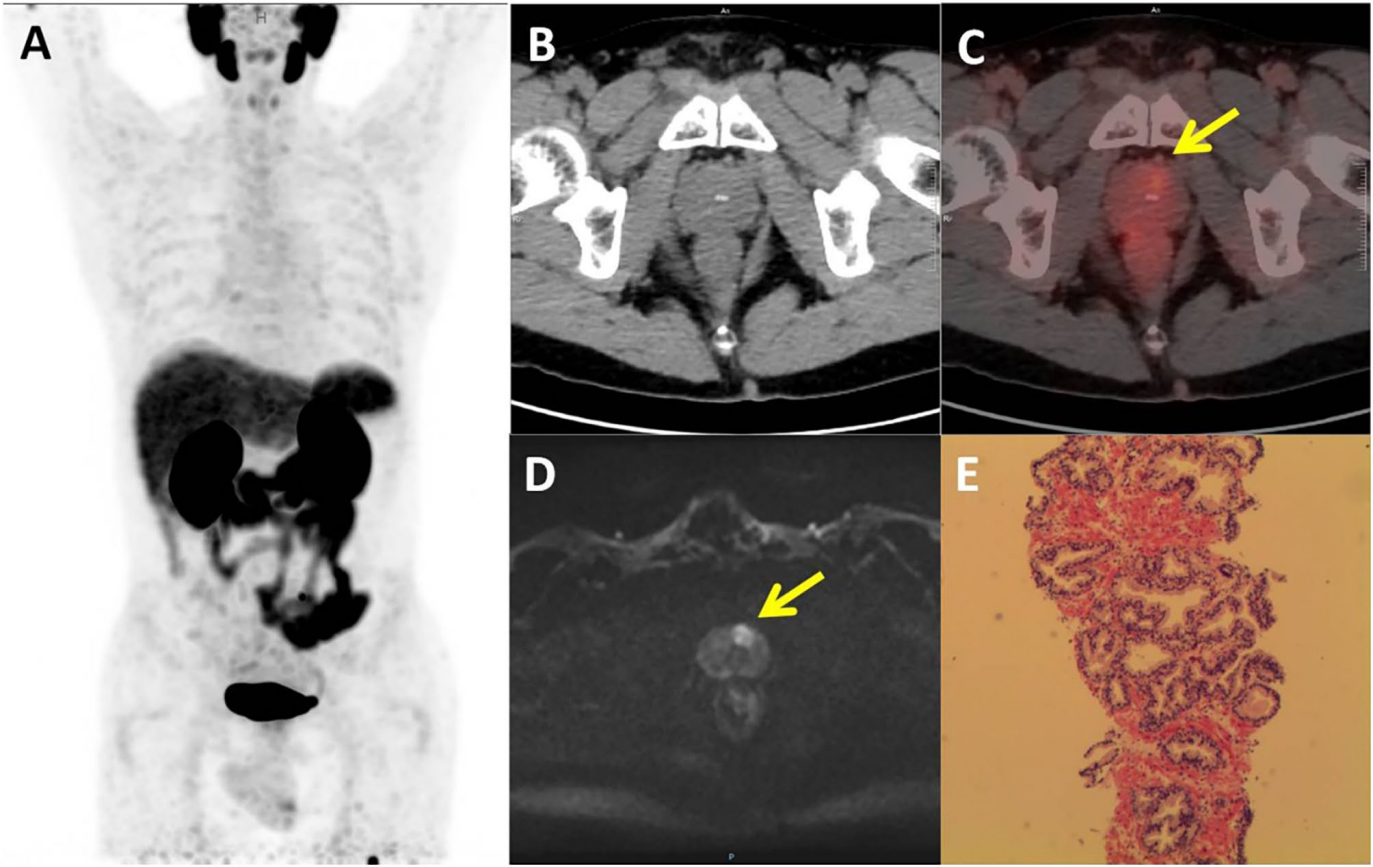
A 63-year-old male with a TPSA level of 7.44 ng/ml. [^18^F]DCFPyL PET/CT (**A**, **B**, **C**) No significant abnormal increase in radiation was observed in the prostate gland, miPSMA score of 1, SUVmax of 3.6. mpMRI Diffusion-Weighted Imaging (DWI) (**D**) The transitional zone at the base of the left prostate shows significant high signal intensity, PI-RADS score of 4. Histopathology (**E**) Nodular hyperplasia of the prostate with slight glandular urethritis changes in the urethral mucosa.

In semi-quantitative analysis, the SUVmax value for the prostate cancer group was 13.1 ± 8.6, significantly higher than the 3.4 ± 1.4 in the benign lesion group (*P* < 0.05). The optimal diagnostic threshold for SUVmax, determined by the Youden index, was 4.17. Using this threshold, [^18^F]DCFPyL PET/CT imaging demonstrated a sensitivity of 92.3%, specificity of 88.2%, positive predictive value of 85.7%, negative predictive value of 93.8%, and accuracy of 90.0%. The typical case is shown in Fig. 1. The AUC from ROC analysis was 0.94. The semi-quantitative analysis of SUVmax values from [^18^F]DCFPyL PET/CT imaging showed high consistency with histopathological results (K = 0.80, *P* < 0.05). All prostate cancer lesions with a PI-RADS score of 4 presented significantly increased metabolic activity, with SUVmax ranging from 4.18 to 27.85, and the semi-quantitative analysis of [^18^F]DCFPyL PET/CT imaging exhibited 100% sensitivity and negative predictive value. One false-negative case had an SUVmax of 3.41 but was later confirmed as prostate cancer with a Gleason score of 3 + 3 and an miPSMA score of 1. Two false-positive cases had SUVmax values of 4.99 and 7.55, but their final pathology confirmed prostate inflammation with miPSMA scores of 1 and 3, respectively.

### Comparing visual and semi-quantitative analyses in [^18^F]DCFPyL PET/CT imaging

**Figure 2:**
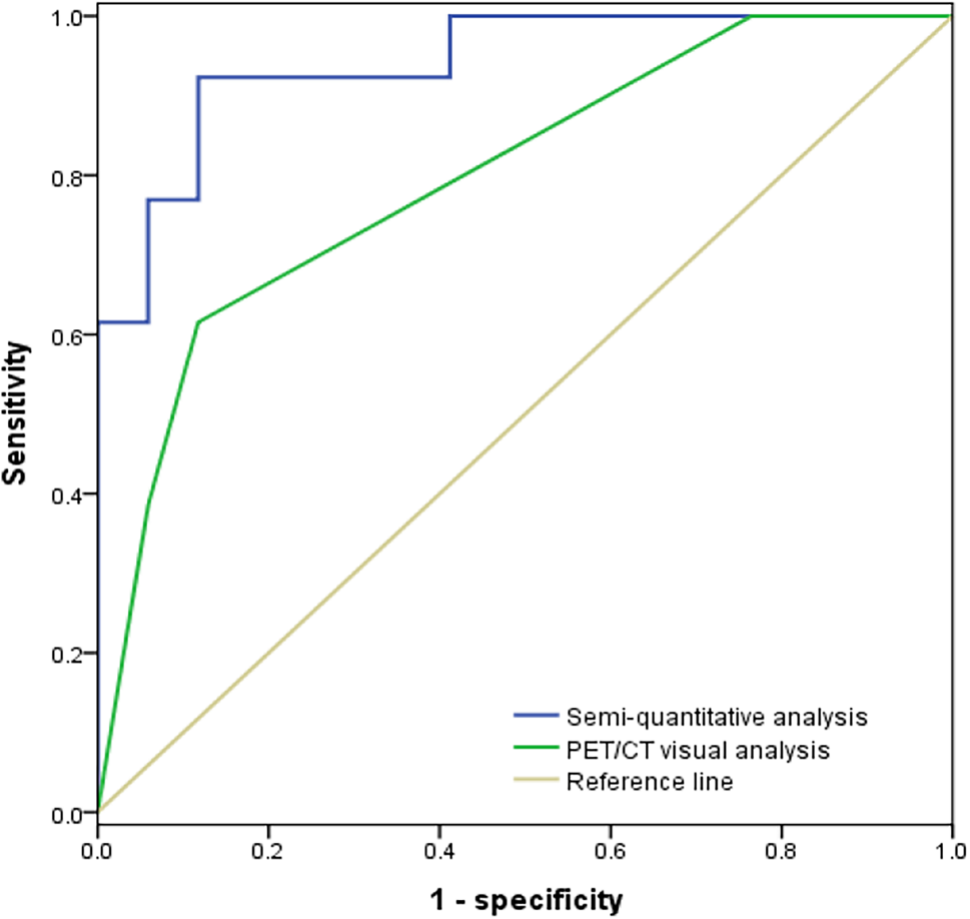
Receiver-operating-characteristic curves of [^18^F]DCFPyL PET/CT Visual and Semi-quantitative analysis.

Comparative analysis of the diagnostic performance of visual and semi-quantitative analyses in [^18^F]DCFPyL PET/CT imaging reveals that semi-quantitative analysis exhibits higher sensitivity, positive predictive value, negative predictive value, and accuracy than visual analysis. However, these differences are not statistically significant (*P* > 0.05). Refer to Table 3 for details. The AUC of the ROC curve in semi-quantitative analysis is significantly larger than that of visual analysis, and the difference is statistically significant (*P* < 0.05). See Fig. 2 for details.

**Table 3:** Efficacy of [^18^F]DCFPyL PET/CT using visual and semi-quantitative analysis.

Index (%)	Visual analysis	Semi-quantitative analysis	*P*
Sensitivity	61.5	92.3	> 0.05
Specificity	88.2	88.2	> 0.05
PPV	80.0	85.7	> 0.05
NPV	75.0	93.8	> 0.05
Accuracy	76.5	90.0	> 0.05

PPV, positive predictive value; NPV, negative predictive value.

## Discussion

Currently, prostate mpMRI is the recommended diagnostic method for preoperative prostate cancer diagnosis, but it still has certain limitations. Particularly for patients with PI-RADS 3/4 scores, mpMRI often struggles to provide a definitive diagnosis, leading to many patients undergoing unnecessary biopsy procedures. In recent years, PSMA-related imaging agents have rapidly developed, gradually replacing traditional ^18^F/^11^C-choline, ^18^F-FDG and ^18^F-fluciclovine, dominating the field of PET diagnosis for prostate cancer. They are widely used for preoperative staging and post-treatment recurrence monitoring.

In this study, for visual analysis, the sensitivity, specificity, positive predictive value, negative predictive value, and accuracy of using miPSMA scoring to diagnose prostate cancer in patients with PI-RADS 3/4 scores were 61.5%, 88.2%, 80.0%, 75.0%, and 76.5%, respectively. In a study by Liu et al.^17^ the sensitivity and specificity for diagnosing prostate cancer using [^18^F]PSMA-7Q PET/CT miPSMA scoring were 91.1% and 83.0%, respectively. The lower sensitivity of [^18^F]DCFPyL PET/CT in our study may be influenced by the inclusion of patients exclusively with PI-RADS 3/4 scores, and the relatively lower proportion of malignant cases (46.4%) in the patient population, leading to more false-negative results. Unlike the [^18^F]PSMA-7Q imaging agent used in Liu Y’s study, the [^18^F]DCFPyL used in our study has a higher liver uptake and a wider uptake range, which may also be an important factor affecting miPSMA scoring diagnosis^17^. And in Laudicella R’s study, cancer lesions are intermingled by benign glands in an infiltrative growth is a potential explanation for lower SUV values of [^68^Ga]Ga-PSMA-11 PET^18^.

In semi-quantitative analysis, for patients with PI-RADS 3/4 scores, malignant lesions had significantly higher SUVmax than benign lesions. When using an SUVmax value of 4.17 as the optimal threshold, the diagnostic sensitivity, specificity, positive predictive value, negative predictive value, and accuracy of [^18^F]DCFPyL PET/CT imaging were 92.3%, 88.2%, 85.7%, 93.8%, and 90.0%, respectively, with an AUC of 0.94. Results from a Mate analysis by Pan KH et al. showed that the sensitivity and specificity of [^18^F]DCFPyL PET/CT for diagnosing prostate cancer were 91% and 90%, respectively, which is consistent with the results of this study^19^. In Zhang T’s study, the sensitivity and specificity of [^18^F]DCFPyL PET/CT for diagnosing prostate cancer were 90% and 100%, with an accuracy of 91.2%, using a diagnostic threshold of SUVmax 5.0^20^. The lower diagnostic threshold in our study is primarily due to the inclusion of patients with PI-RADS 3/4 scores. Additionally, only 69.2% of patients in this study had a Gleason score of ≥ 7, whereas in Zhang T’s study, there was no explicit limitation on the inclusion of patients based on PIRADS scores, and a higher proportion of patients were malignant, with a higher Gleason score (≥ 7) of 87.5%.

In the comparative analysis of diagnostic performance between visual analysis and semi-quantitative analysis, it was observed that semi-quantitative analysis exhibited higher sensitivity, positive predictive value, negative predictive value, and accuracy than visual analysis. However, due to the limited number of cases in this study, these indicators did not show statistically significant differences. By constructing ROC curves and comparing the AUC, it was found that the AUC of the semi-quantitative analysis was significantly higher than that of visual analysis in diagnosing prostate cancer. Furthermore, the application of semi-quantitative analysis showed better consistency with histopathological results. In visual analysis, there were four cases of false-negative results for prostate cancer, all of which were accurately diagnosed in the quantitative analysis. This suggests that visual analysis can still be influenced by the metabolic levels of various organs, including the blood pool, liver, and salivary glands. Eiber M et al. suggested that miPSMA alone cannot diagnose prostate cancer and that clinical context and other imaging results need to be considered^16,21^. In Derwael C’s study, although different radiologists showed good consistency in miPSMA scoring in [^68^Ga]Ga-PSMA-11 PET/CT imaging for newly diagnosed prostate cancer patients, the authors still acknowledged that the accuracy of this scoring was influenced by the uptake in the prostate gland, lymph nodes, and bones^22^.

In this study, among the PI-RADS 3/4 score patients included, 17 cases (56.7%) were diagnosed with benign prostate conditions. Through [^18^F]DCFPyL PET/CT imaging, 15 of these patients had SUVmax values ranging from 1.48 to 4.15, which were below the diagnostic threshold, indicating benign lesions. These patients could avoid unnecessary biopsy examinations, accounting for 50% of the total patients. Research by Yang J et al. found that compared to mpMRI and clinical presentation, [^68^Ga]Ga-PSMA-11 PET/CT demonstrated higher diagnostic efficacy for PI-RADS 3 score patients and had an advantage over the european randomized study of screening for prostate cancer risk calculator 3 (ERSPC-RC3) and prostate cancer prevention trial risk calculator (PCPT-RC) models^23^. In a study by Wong LM et al. involving PI-RADS 4/5 score patients, [^18^F]DCFPyL PET/CT showed a good negative predictive value of 93.3%, allowing patients with negative imaging to safely avoid prostate biopsies^24^. There were two cases of benign lesion patients in [^18^F]DCFPyL PET/CT imaging who exhibited false-positive results, one with PI-RADS 3 score and the other with a 4 score, with maximum SUV values of 4.99 and 7.55, respectively. These cases were ultimately confirmed as local chronic prostate inflammation. Previous studies have suggested that the increased uptake of [^18^F]DCFPyL may be associated with the increased neovascularization in prostate inflammatory lesions^25^. Therefore, when analyzing positive lesions in prostate [^18^F]DCFPyL PET/CT imaging, it is essential to consider comprehensive diagnostic factors such as PSA levels and clinical presentation. Among patients with a PI-RADS score of 3, there was one case of prostate cancer where [^18^F]DCFPyL PET/CT imaging yielded a false-negative result with an SUVmax of 3.41. This patient’s final pathological Gleason score was 3 + 3, and ISUP grading was 1. The well-differentiated nature of this lesion, along with relatively low PSMA expression, led to the false-negative result. Clinical studies have shown that patients with a Gleason score of 3 + 3 often have a favorable prognosis. There is still significant debate regarding the treatment approach for these patients. Currently, most prostate cancer treatment guidelines worldwide recommend an observation-based approach, primarily active surveillance, for such cases, resembling a treatment model for precancerous conditions rather than curative treatment for the majority of cancers^26,27^. In the 12 cases with a PI-RADS score of 4, all patients displayed positive findings in [^18^F]DCFPyL PET/CT imaging, with SUVmax values ranging from 4.18 to 27.85, resulting in 100% sensitivity and negative predictive value. A study by Emmett et al., which combined mpMRI and [^68^Ga]Ga-PSMA-11 PET/CT imaging for patients with PI-RADS ≥ 4 scores, showed that the specificity and positive predictive value for detecting prostate cancer were both 100%^28^. Therefore, we believe that non-invasive radical prostatectomy may be a feasible treatment in this group of patients.

Growing evidence proved the efficacy of PSMA PET/CT-guided targeted biopsy in prostate cancer diagnosis. In this study, [^18^F]DCFPyL PET/CT showed a good negative predictive value. A negative [^18^F]DCFPyL PET/CT in men with equivocal or positive mpMRI with other concerning features prompting biopsy, [^18^F]DCFPyL PET/CT prior to biopsy may facilitate targeted biopsy to improve diagnostic accuracy. [^18^F]DCFPyL PET/CT used in this context may reduce unnecessary biopsies and their complications, as well as decrease the diagnosis of low grade, indolent disease. In Ferraro DA’s study, five patients with suspected prostate cancer underwent [^18^F]PSMA-1007 PET/CT scans followed by immediate PET/CT-guided and saturation template biopsy. Prostate cancer was present in 43 of 113 needles, and the mean counts per minute (cpm) was overall significantly higher in needles with prostate cancer compared to needles without prostate cancer^29^. This study could improve confidence in imaging-based biopsy guidance and reduce the need for saturation biopsy.

This study has some limitations. Firstly, this study is a retrospective, single-center study with a small patient sample, which limits the accuracy of the results and the generalizability of the study findings. Another limitation is that the pathology reference standards for patients were established through biopsies, which may carry a risk of misclassification and lower clinical significance in detecting prostate cancer compared to prostatectomy specimens.

## Conclusion

[18F]DCFPyL PET/CT imaging provides potential preoperative prediction of lesion nature in mpMRI PI-RADS 3/4 patients, which may aid in treatment decision-making and reducing unnecessary biopsies.

### Supplementary Information

Supplementary Information.

## Data Availability

Data is provided within the manuscript or supplementary information files.
